# Superficial Laceration of the Female Perineum

**Published:** 1893-10

**Authors:** Frederick P. Hammond

**Affiliations:** New York City


### Selections.

---

### SUPERFICIAL LACERATION OF THE FEMALE PERINEUM.

By FREDERICK P. HAMMOND, M. D., New York City.

In the evolution of research concerning the female perineum and its diseases, a few suggestions do not seem out of place concerning the pathological changes which take place following a laceration of the skin and superficial connective tissue. Our older teachers and many of the present day deal with the area of tissue between the vagina and rectum as the perineal body, and teach that its main function is the support of the female pelvic viscera. The newer school, unable to believe Nature had been so imperfect in her work as to leave the important organs of generation dependent upon a mass of connective tissue and skin for their support, sought for and found the more logical pelvic floor, consisting chiefly of the levator ani and triangular ligament. But these, having found the true support of the pelvic viscera, have in their enthusiasm lost sight of this still important organ, the perineal body. For, in a degree, it still remains a body, and its function is hardly less important than our older teachers held, for it closes and protects the vaginal outlet from the atmosphere and friction of the clothing, and affords support for the superficial perineal muscles.

From a careful observation among dispensary patients and in my private practice, I have taken special note of the existing condition of affairs in all cases presenting themselves where a laceration of the superficial perineum, or relaxation of the pelvic floor, due to a division of the fibers of the levator ani, has existed, and in both the resulting pathological condition has been the same.

From my observation, therefore, it seems conclusive that the more modern teaching of the slight laceration of the superficial perineum being harmless, further than for its cosmetic effect, is entirely erroneous. For, if any gynecologist wishes to convince himself that hypertrophy and edema of the vaginal walls follow a superficial laceration as well as a relaxed pelvic floor, he has ample opportunity in observing a number of the cases presenting themselves before him. While I admit that the serious train of symptoms, such as cystocele and rectocele, with its attendant prolapse and subjective symptoms, is delayed in a case of simple laceration, it as certainly results from a case of relaxation. For, given any case, where the normal protection from atmospheric irritation and friction of the clothing is destroyed, hypertrophy and edema are the logical result. And while those with opposite views would combat this argument with the circumstance that they have observed cystocele and rectocele in virgins who have passed the menopause, they must remember that they cite one of the rare occurrences of pathology, and might be brought to see the absurdity of their views by going still further and calling to mind the fact that cases of complete prolapsus uteri have occurred in strong, robust domestics, who have the hymen intact.

The sooner we, in a degree, come back to a realization of the fact that a laceration of the perineum, however slight, is a pathological condition which demands interference as much as any of the other ailments of the female genital tract, the earlier shall we relieve our patients of so much suffering. There are many today who, for a case of endometritis or salpingitis, advocate dilatation of the os and curetting—an operation not less serious in its results than a salporrhaphy in the hands of an ordinarily careful operator. Why, then, do we remain as extremists in our views regarding this condition, when proof is forthcoming that in this, as with nearly all others where minds have been at variance, a middle ground is our shield and haven ?—*Medical Record.*

